# Effects of a Tart Cherry Supplement on Recovery from Exhaustive Exercise

**DOI:** 10.3390/jfmk8030121

**Published:** 2023-08-18

**Authors:** Dolores G. Ortega, Jared W. Coburn, Andrew J. Galpin, Pablo B. Costa

**Affiliations:** Exercise Physiology Laboratory, Department of Kinesiology, California State University, Fullerton, CA 92831, USA

**Keywords:** polyphenols, antioxidants, muscle soreness, muscle function, recovery, resistance exercise

## Abstract

The aim of this study was to investigate the effects of a tart cherry supplement on recovery from exercise-induced muscle damage. Seventeen recreationally active women (mean age ± SD = 22.2 ± 3.3 years, height = 162.0 ± 6.0 cm, body mass = 65.1 ± 11.1 kg, BMI = 24.7 ± 3.5 kg·m^2^) supplemented with 1000 mg of concentrated tart cherry or a placebo for eight consecutive days. An overload protocol of 8 sets of 10 repetitions of maximal effort concentric and eccentric muscle actions of the leg extensors at a velocity of 60${}^{\circ}\cdot s^{-1}$ was performed on the fourth day of supplementation. Testing sessions consisted of a muscle function test (MFT) to examine pre- and post-testing peak torque, peak power, total work, time-to-peak torque, mean power, muscle activation of the quadriceps, and muscle soreness at baseline and post-testing at 0 h, 24 h, 48 h, and 72 h. A second trial of testing was repeated two weeks later using the opposite supplement to the one assigned for the first trial. No significant interaction for time × condition × velocity (*p* = 0.916) and no significant main effect for condition (*p* = 0.557) were demonstrated for peak torque. However, there were main effects for time and velocity for concentric quadriceps peak torque (*p* < 0.001). For muscle soreness, there was no two-way interaction for time x condition (*p* > 0.05) and no main effect for condition (*p* > 0.05), but there was a main effect for time (*p* < 0.001). In conclusion, a tart cherry supplement did not attenuate losses in isokinetic muscle peak torque, peak power, total work, time-to-peak torque, muscle soreness, or quadriceps muscle activation.

## 1. Introduction

Resistance exercise is commonly used for muscle strength and muscle mass gains to improve athletic performance or decrease the demands of activities of daily living [1,2]. However, unaccustomed, strenuous, high repetition eccentric training produces higher incidences of exercise-induced muscle damage (EIMD) [1,2,3,4,5,6,7,8,9]. EIMD may arise due to mechanical stress or metabolic overload; however, its development is not completely understood [6,7,8]. The mode, intensity, and duration of exercise, as well as the training experience of an individual determine the severity of EIMD [10]. Common symptoms associated with EIMD include a reduction in muscle force, an increased inflammatory response in the bloodstream, a reduction in range of motion (ROM), a reduction in neuromuscular function, an increase in limb circumference due to swelling, and the development of delayed-onset muscle soreness (DOMS) [6,7,8]. Direct measurement of EIMD markers is invasive and includes blood draws to measure plasma creatine kinase and protein concentrations [11]. Thus, indirect measures of EIMD include increased muscle soreness, a reduction in joint ROM, increased tissue swelling, and loss of muscle strength [11,12]. Accordingly, DOMS and muscle strength are the most notable indirect markers used to evaluate EIMD [13]. Potential recovery methods following exercise training would be beneficial, since the presence of DOMS can decrease exercise tolerance and increase the perception of effort, which may then alter athletic performance or adherence to an exercise training program [6].

A variety of recovery methods to attenuate EIMD have been suggested and can be divided into three categories: (1) therapeutic, (2) pharmacological, and (3) nutritional interventions [12]. Passive stretching, foam rolling, and vibrating foam rolling are therapeutic interventions, which have been previously researched [14,15,16,17]. Meanwhile, non-steroidal anti-inflammatory drugs (NSAIDs) are an example of a pharmacological intervention used to treat muscle damage [12,14]. Nutritional interventions are an alternative to therapeutic and pharmacological interventions [12,14]. Exercise can cause oxidative stress, which occurs when there is an imbalance between reactive oxygen species (ROS) production and antioxidant defenses [18]. Thus, antioxidant supplementation has gained popularity for its proposed ability to inhibit ROS production and reduce muscle damage following intense exercise [14,19]. Previous studies have examined the effects of multi-ingredient supplements on exercise performance [20,21]. For example, it was reported that a combined antioxidant and protein supplement resulted in less muscle soreness following eccentric exercise than a protein or carbohydrate supplement alone [20]. Supplementation with antioxidants, however, may have a detrimental effect on skeletal muscle adaptations to resistance training [22].

Tart cherries are dietary supplements of interest, since they are polyphenol-rich foods, which may promote recovery from EIMD with their antioxidant and anti-inflammatory properties [7]. Flavonoids, a subclass of polyphenols, and anthocyanins, a subclass of flavonoids, have garnered the most interest in research [23]. Specifically, the properties of anthocyanins are understood to increase the expression of endogenous antioxidant enzymes while scavenging ROS and limiting their production [8]. The mode of exercise, however, may influence the efficacy of tart cherry supplementation on symptoms of EIMD [24]. Mixed results have been reported in research assessing the efficacy of tart cherry supplementation on recovery following plyometric exercise and resistance training [2,3,4,6,7,25,26,27]. In particular, two studies have examined the effects of tart cherry following eccentric training of the knee extensor muscles [3,4]. Beals et al. [3] reported that a tart cherry beverage containing 733 mg of phenolic compounds ingested for 12 days did not reduce the pain and muscle tenderness associated with DOMS following a knee extension eccentric training protocol on a Biodex Multi Joint System among resistance trained men aged 18–50 years old. In contrast, the results of Bowtell et al. [4] indicated supplementing with 30 mL of cherry juice concentrate for 10 days improved recovery of isometric knee extension muscle strength among well-trained males between 25 and 29 years of age. Interestingly, most of the research to date has used men or mixed-sex populations [28]. 

Tart cherry supplementation in powdered form and its effects on isokinetic performance and recovery among recreationally active college-aged women have not been researched. Therefore, the purpose of this study was to investigate the effects of a tart cherry supplement on recovery from exhaustive exercise as measured via isokinetic muscle peak torque, isokinetic muscle work, isokinetic muscle peak power, isokinetic mean power, muscle soreness, and muscle activation. It was hypothesized that tart cherry supplementation would improve recovery following an intense bout of resistance exercise measured via attenuated losses in isokinetic muscle peak torque, peak power, total work, time-to-peak torque, mean power, and muscle activation. It was also hypothesized that tart cherry supplementation would attenuate muscle soreness.

## 2. Methods

### 2.1. Subjects

Seventeen women participated in this study (mean age ± SD = 22.18 ± 3.32 years, body mass = 65.11 ± 11.08 kg, height = 162.03 ± 6.02 cm, BMI = 24.71 ± 3.52 kg ·m^2^). Participants were recreationally active and of collegiate age. Recreationally active was defined as participating in physical activity at least 3 days per week, for at least 30 min each session, for at least 3 months [6]. Participants were free of any lower extremity injury within the last six months of their participation in the study [29]. Participants had no reported aversion or inability to tolerate intense, soreness-inducing resistance training exercises or a history of medical events (i.e., exercise-induced rhabdomyolysis, cardiovascular disease, metabolic, renal, hepatic, or musculoskeletal disorders), which may have significantly affected the study outcome [3]. In addition, participants did not use medicine or nutritional supplements, which may have affected the study outcome for one month prior to the onset of data collection [3]. During the loading phases of this study, participants were asked to refrain from consuming supplemental protein or branched-chain amino acids in quantities greater than two servings per week, antioxidant or anti-inflammatory supplements or drugs, grapefruit and grapefruit juice, steroids, caffeine, marijuana, and alcohol [7,26,28]. Furthermore, participants were asked to not engage in treatments to aid with muscle soreness [28], such as massage, foam rolling, topical analgesics, or ice therapy. Lastly, participants were asked to refrain from partaking in any lower body exercise throughout the duration of the study [26].

### 2.2. Research Design

**Figure 1:**
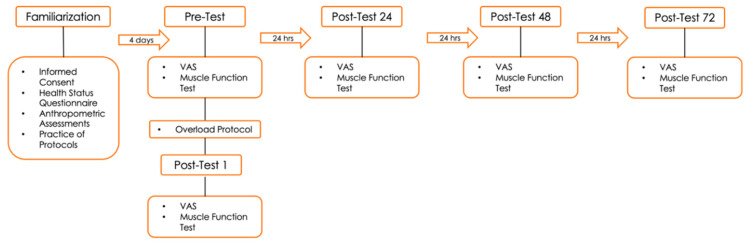
Description of each session.

The study utilized a randomized, double-blind, placebo-controlled crossover design. Participants visited the laboratory on nine separate occasions (Figure 1). The first visit was a familiarization session, where participants completed the informed consent form, filled out a pre-exercise testing health and exercise status questionnaire, and set a schedule for the remaining laboratory visits. Participants were also familiarized with the isokinetic dynamometer, electromyography (EMG) electrodes, and the testing environment by completing a preliminary trial of the muscle function test (MFT) and overload protocol. Once that was completed, each participant was randomly assigned the first of two de-identified “supplements” (tart cherry supplement or placebo) and was instructed to begin taking the supplement on this day to mark the start of Trial 1 and day 1 of the loading phase. They were asked to consume this supplement daily for a 4-day loading phase. The loading phase was followed by Visit 1, during which the participant underwent an overload protocol consisting of a series of muscle actions of the randomly selected quadriceps in an isokinetic dynamometer. Participants continued to consume the supplement on the day of the overload protocol and 24, 48, and 72 h after overload. Testing occurred at pre-overload and 0 (immediately after), 24, 48, and 72 h after overload. Testing consisted of isokinetic muscle peak torque, isokinetic muscle work, and isokinetic muscle power using 3 concentric/concentric leg extension repetitions at speeds of 60°·$s^{-1}$, 180°·$s^{-1}$, and 300°·$s^{-1}$, as well as muscle activation using EMG. The overload protocol consisted of 8 sets of 10 concentric/eccentric leg extension repetitions at 60°·$s^{-1}$. A 14-day wash-out began after the completion of Trial 1 testing, and the 4-day loading phase of the next product occurred after the wash-out. All protocols were repeated on the contralateral lower limb for the second product to avoid any potential remaining impact of the repeated bout effect [26].

### 2.3. Procedures

Body mass was measured using a digital scale (Ohaus ES Series scale, Parsippany, NJ, USA), and height was measured using a stadiometer (SEXA stadiometer, Chino, CA, USA). Participants were then familiarized with EMG electrode locations and the isokinetic dynamometer by completing a preliminary MFT trial and a set of 10 repetitions of the overload protocol. The preliminary MFT trial consisted of three maximal effort leg extension repetitions at three different velocities (60°·$s^{-1}$, 180°·$s^{-1}$, and 300°·$s^{-1}$) after a separate warm-up for each velocity. The overload protocol preliminary trial consisted of 1 set of 10 leg extension repetitions at 60°·$s^{-1}$ to reduce any potential learning effects, since the preceding visits were testing days. At the end of the familiarization visit, a 10-point visual analog scale (VAS)—which was used to measure muscle soreness while at rest, walking downstairs, and performing a squat—was explained to each participant. Participants were then assigned to one of the two de-identified supplements and asked to begin the 4-day loading phase and visit the lab on day 5 of supplementation to perform the overload protocol.

On Visit 1 of Trial 1, participants completed three separate parts. The first part was a pre-test, which began with baseline VAS and MFT performed via the isokinetic dynamometer on the randomly assigned quadriceps to establish muscle soreness, concentric peak torque, concentric peak power, concentric mean power, concentric time-to-peak torque, concentric total work, and muscle activation. EMG electrodes were used to detect and assess muscle activation. The participant was then asked to remain on the isokinetic dynamometer to complete the overload protocol to elicit the quadriceps muscle overload. Immediately after the overload protocol, participants repeated the VAS and MFT to assess muscle soreness, peak torque, peak power, mean power, time-to-peak torque, total work, and muscle activation. Visits 2, 3, and 4 correlated with post-testing at 24 h, 48 h, and 72 h. The participant was asked to complete a post-VAS muscle soreness rating and post-MFT on each of those visits. Trial 1 was completed in 8 days.

After Visit 4, participants commenced a 14-day wash-out phase and were asked to continue to refrain from any prohibited supplementation or activity. The participants arranged a time and day to acquire the second supplement to prepare for Trial 2. Trial 2 was then a repeat of the procedures outlined for Trial 1. The participants underwent a loading phase with the second de-identified supplement, and the contralateral lower limb underwent the overload protocol and was tested.

At the same time as testing, participants were asked to record a 5-day food, exercise, and sitting log using MyFitnessPal (MyFitnessPal, Inc., San Francisco, CA, USA)—an application and web-based dietary tracking system. Subjects were then asked to replicate diets from the initial Trial 1 in Trial 2.

### 2.4. Supplementation Protocol

Participants began consuming the first of the two randomly assigned de-identified supplements after the familiarization visit. Each participant consumed the supplement four days prior to the overload protocol, on the day of the overload protocol, and three days after the overload protocol. Previous research had participants consume a supplement for a total of eight days [19,25,27,28,30,31]. Daily polyphenol supplementation for three or more days before and after exercise has been shown to improve recovery [2]. The experimental tart cherry supplement used in this study was highly concentrated tart cherry capsules (Toniiq LLC, Chicago, IL, USA). Each participant consumed two capsules containing a total of 1000 mg concentrated tart cherry extract for a total of eight days. For the placebo, subjects consumed two capsules of dextrose and natural red food coloring (Muscle Feast, Nashport, OH, USA), which resembled the tart cherry capsules, for a total of eight days. A 14-day wash-out phase was completed in between each trial [2].

### 2.5. Muscle Function Test Protocol

The MFT protocol was completed using an isokinetic dynamometer (Humac Norm CSMi, Stoughton, MA, USA) to analyze peak torque, peak power, mean power, time-to-peak torque, and total work at the velocities of 60${}^{\circ}\cdot s^{-1}$, 180${}^{\circ}\cdot s^{-1}$, and 300${}^{\circ}\cdot s^{-1}$ [29,32,33]. Isometric leg extension MVC was measured at 50% of each participant’s leg extension range of motion (ROM) to establish the MVCs for EMG normalization [33]. Subjects were seated on the isokinetic dynamometer, and straps were fastened over the participants’ shoulders, across their lap, and around the shin of the testing leg to ensure it was isolated and secured [33]. The axis of the dynamometer was aligned to meet the knee rotation axis of the secured leg [29,33]. Each MFT session began with warm-up kicks and pulls at increasing intensities of 25%, 50%, 75%, and 100%, followed by a 1 min rest period [29,32,33]. Participants performed three maximal effort repetitions of the concentric extension and flexion actions at the three different velocities, and the highest value of each of the three maximal repetitions was recorded [33]. All other dependent variables were recorded based on the best repetition at each velocity. A 1 min rest period was provided between each velocity [33]. Participants were given verbal prompts and encouragement, such as “kick”, “pull”, and “push” [29,33]. 

### 2.6. Electromyography (EMG) Protocol

EMG electrodes were placed on the participant’s testing limb prior to performing the MFT and overload protocol to measure muscle activation. Two pre-amplified bipolar surface electrodes (EL254S; Biopac Systems Inc., Santa Barbara, CA, USA) were placed over the rectus femoris (RF) and vastus lateralis (VL) of the quadriceps [29,33]. The electrode on the RF was placed at the mid-point of the anterior superior iliac spine and the superior part of the patella [29]. The electrode on the VL was placed at 2/3 the distance between the anterior superior iliac spine and the lateral portion of the patella [33]. To ensure the EMG electrode placements were identical for each session, the RF and VL EMG locations were marked during the first day of each de-identified supplement trial. The reference electrode was placed over the spinous process of the seventh cervical vertebra [29,33]. The electrodes were placed on the skin after shaving, abrading, and cleaning with isopropyl alcohol at each session [29,33]. Raw EMG scores of muscle activation for the two muscles were collected using a Biopac data collection system (MP150WSW; Biopac Systems Inc, Santa Barbara, CA, USA). All EMG signals were recorded at a frequency of 1000 (hertz) Hz during the completion of the MFT. EMG values were filtered with signal bandpass at 10–500 Hz, and data were measured and recorded as root mean square and normalized to the maximum voluntary contractions (MVCs). All signals were recorded on a personal computer (Dell, Red Rock, TX, USA) and analyzed using AcqKnowledge (version 5.0, Biopac Systems Inc., Santa Barbara, CA, USA).

### 2.7. Overload Protocol

The overload protocol used to induce muscle damage was completed using anisokinetic dynamometer (Humac Norm CSMi, Stoughton, MA, USA). The protocol consisted of 8 sets of 10 repetitions of maximal effort leg extensions with concentric and eccentric quadriceps muscle actions at a velocity of 60${}^{\circ}\cdot s^{-1}$ with 1 min rest periods in between sets. Beals et al. [3] had participants perform concentric/eccentric knee extension contractions at 60${}^{\circ}\cdot s^{-1}$ using sets of 45, 45, and 90 repetitions but found no significant increase in CK, indicating the protocol may not have induced enough muscle damage to produce an inflammatory response [3]. This protocol intended to elicit significant quadriceps muscle damage using a higher number of sets with a lower number of repetitions per set, as suggested by previous research [3]. 

### 2.8. Visual Analog Scale (VAS) Protocol

Quadriceps muscle soreness was assessed using a 10-point VAS. A rating of 0 on the VAS signified no soreness, while a rating of 10 signified worst possible soreness [33]. For this test, participants were asked for a rating of soreness while resting [6], walking downstairs [34,35], and performing a squat [6,8]. The resting VAS measure was assessed while the participant was seated on a chair. The walking downstairs VAS was recorded while the participant walked down 13 steps with a height of 20.5 cm, width of 30.4 cm, and length of 42 cm. To perform the squat, each participant stood with their hands on their hips and feet shoulder-width apart, squatted to 90° while flexing at the knees, and then stood back to the start position [6]. The VAS has been shown to be a reliable method of assessing perceived muscle pain after eccentric exercise [36].

### 2.9. Statistical Analysis

Peak torque, peak power, mean power, time-to-peak torque, total work, RF EMG, and VL EMG were analyzed using a three-way repeated-measures ANOVA (time [pre vs. 0 h post vs. 24 h post vs. 48 h post vs. 72 h post] × condition [placebo vs. supplement] × velocity [60°·$s^{-1}$ vs. 180°·$s^{-1}$ vs. 300°·$s^{-1}$]). Muscle soreness ratings were analyzed through a two-way repeated-measures ANOVA (time [pre vs. 0 h post vs. 24 h post vs. 48 h post vs. 72 h post] × condition [placebo vs. supplement]). Post hoc one-way ANOVAs and *T*-tests with a Bonferroni correction were used if necessary and appropriate. Data were reported as mean ± *SE*. The results were considered significant at *p* ≤ 0.05. IBM SPSS Statistics was used for statistical analysis (version 28, IBM Corp, Armonk, NY, USA).

## 3. Results

### 3.1. Peak Torque

**Figure 2:**
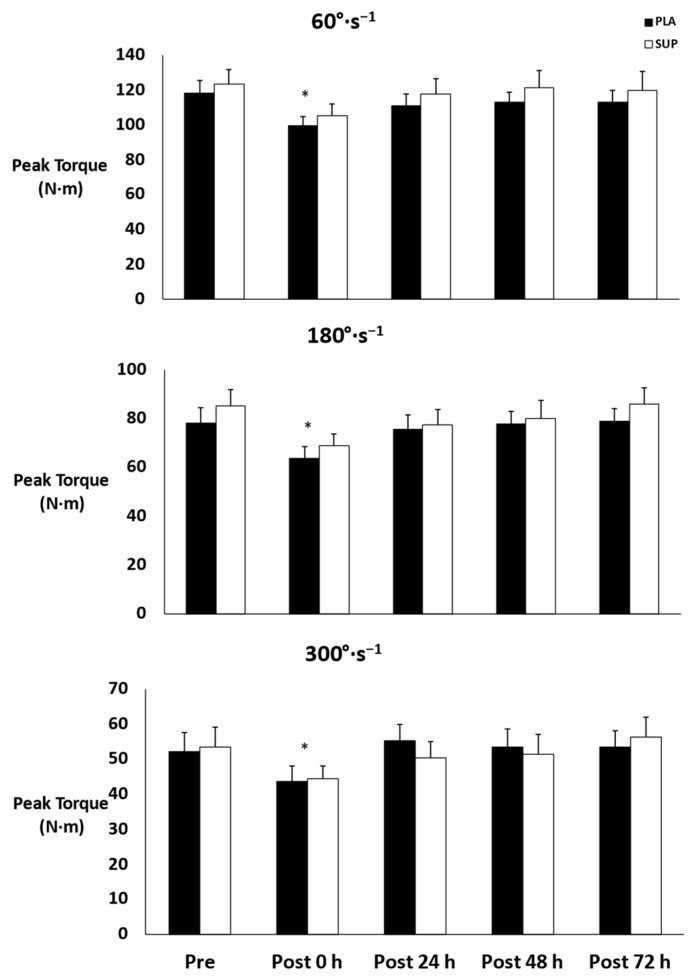
Mean ± SE of peak torque. Pre: Pre-test. Post 0 h: Post-test 0 h. Post 24 h: Post-test 24 h. Post 48 h: Post-test 48 h. Post 72 h: Post-test 72 h. PLA: Placebo. SUP: Tart Cherry Supplement. * Denotes significant difference from pre- and post-test 24, 48, and 72 h.

There was no three-way interaction for time × condition × velocity (*p* = 0.916) and no two-way interactions for time × condition (*p* = 0.853), time × velocity (*p* = 0.157), or condition × velocity (*p* = 0.114). However, there were main effects for time and velocity (*p* < 0.001). Peak torque decreased from pre- to post-test 0 h (*p* < 0.001) and increased post-test 24 h (*p* < 0.001), post-test 48 h (*p* = 0.011), and post-test 72 h (*p* = 0.007) from post-test 0 h (Figure 2). In addition, peak torque decreased as angular velocity increased (*p* < 0.001).

### 3.2. Peak Power 

**Figure 3:**
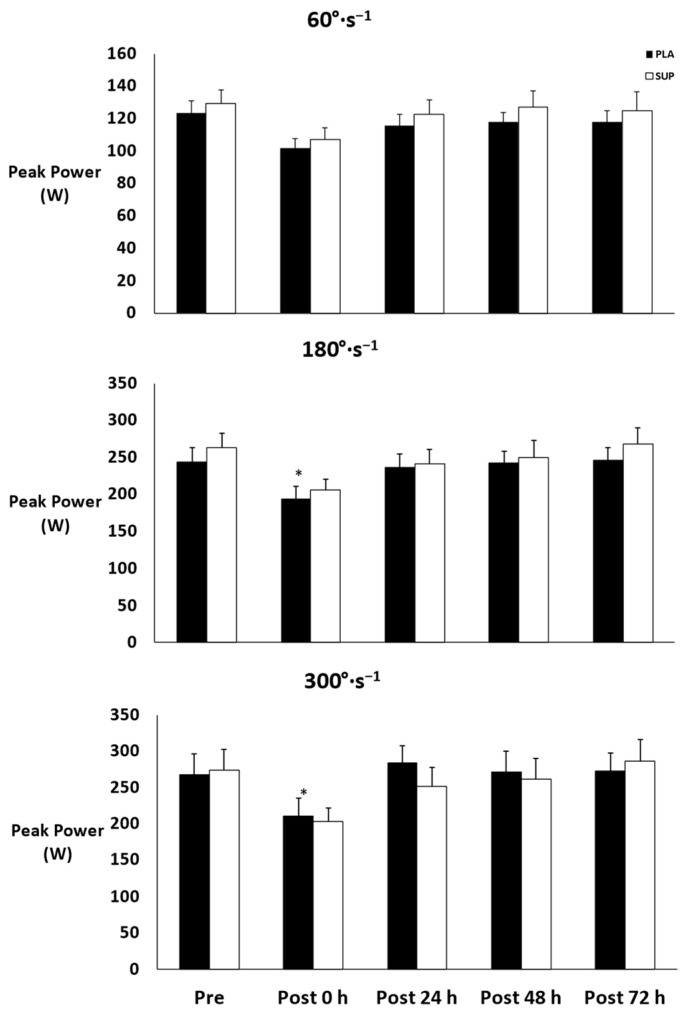
Mean ± SE of peak power. Pre: Pre-test. Post 0 h: Post-test 0 h. Post 24 h: Post-test 24 h. Post 48 h: Post-test 48 h. Post 72 h: Post-test 72 h. PLA: Placebo. SUP: Tart Cherry Supplement. * Denotes significant difference from pre- and post-test 24, 48, and 72 h.

There was no three-way interaction for time × condition × velocity (*p* = 0.648) and no two-way interactions for time × condition (*p* = 0.665) or condition × velocity (*p* = 0.258). However, there was a two-way interaction for time × velocity (*p* < 0.001). Simple main effects were found for 180°·$s^{-1}$ and 300${}^{\circ}\cdot s^{-1}$ (*p* < 0.001) but not for 60°·s^−1^ (*p* = 0.421). At 180${}^{\circ}\cdot s^{-1}$, peak power decreased from pre- to post-test 0 h (*p* = 0.018) and increased at post-test 24 h (*p* = 0.039), post-test 48 h (*p* = 0.011), and post-test 72 h (*p* < 0.001) from post-test 0 h. At 300${}^{\circ}\cdot s^{-1}$, peak power decreased from pre- to post-test 0 h (*p* = 0.016) and increased at post-test 24 h (*p* = 0.004), post-test 48 h (*p* = 0.009), and post-test 72 h (*p* < 0.001) from post-test 0 h (Figure 3).

### 3.3. Total Work

**Figure 4:**
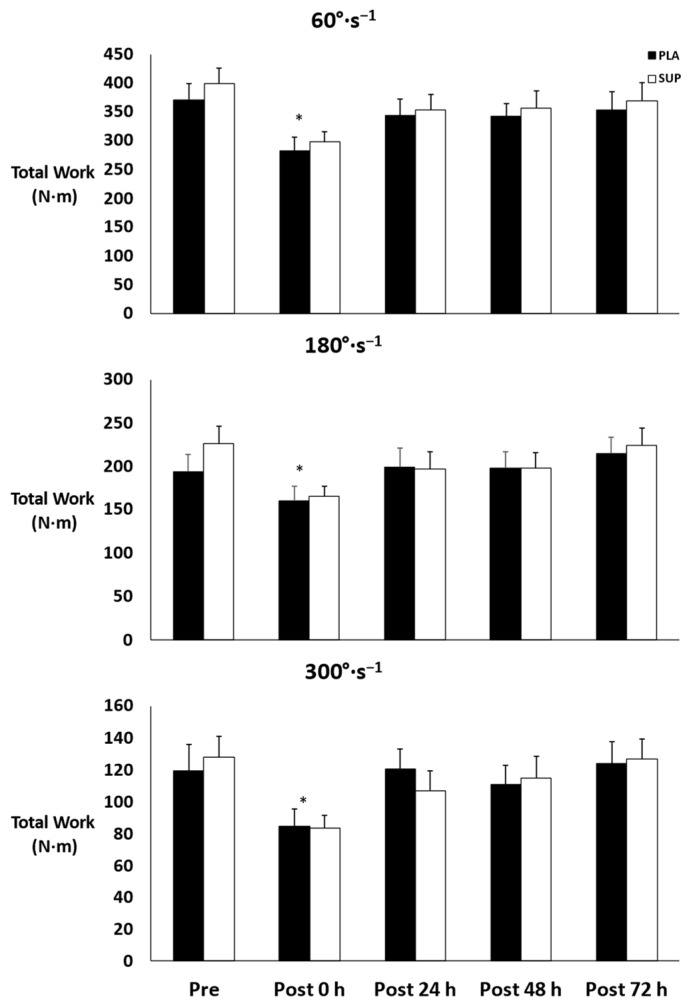
Mean ± SE of total work. Pre: Pre-test. Post 0 h: Post-test 0 h. Post 24 h: Post-test 24 h. Post 48 h: Post-test 48 h. Post 72 h: Post-test 72 h. PLA: Placebo. SUP: Supplement. * Denotes significant difference from pre- and post-test 24, 48, and 72 h.

There was no three-way interaction for time × condition × velocity (*p* = 0.951) and no two-way interactions for time × condition (*p* = 0.473) or condition × velocity (*p* = 0.456). However, there was a two-way interaction for time × velocity (*p* = 0.001). Simple main effects were found for all three velocities (*p* < 0.001). At 60°·s^−1^, total work decreased from pre- to post-test 0 h (*p* < 0.001) before increasing at post-test 24 h (*p* = 0.009), post-test 48 h (*p* = 0.011), and post-test 72 h (*p* = 0.015). At 180${}^{\circ}\cdot s^{-1}$, total work decreased from pre- to post-test 0 h (*p* = 0.020) and increased at post-test 24 h (*p* = 0.009), post-test 48 h (*p* = 0.005), and post-test 72 h (*p* < 0.001) from post-test 0 h. At 300${}^{\circ}\cdot s^{-1}$, total work decreased from pre- to post-test 0 h (*p* = 0.020) and increased at post-test 24 h (*p* < 0.001), post-test 48 h (*p* = 0.003), and post-test 72 h (*p* < 0.001) from post-test 0 h (Figure 4).

### 3.4. Time-to-Peak Torque

**Figure 5:**
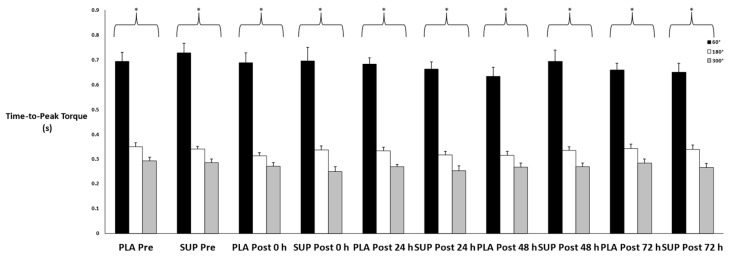
Mean ± SE of time-to-peak torque. Pre: Pre-test. Post 0 h: Post-test 0 h. Post 24 h: Post-test 24 h. Post 48 h: Post-test 48 h. Post 72 h: Post-test 72 h. 60°: Velocity of 60${}^{\circ}\cdot s^{-1}$. 180°: Velocity of 180${}^{\circ}\cdot s^{-1}$. 300°: Velocity of 300${}^{\circ}\cdot s^{-1}$. * Denotes significant difference among velocities.

There was no three-way interaction for time × condition × velocity (*p* = 0.779) and no two-way interactions for time × condition (*p* = 0.498), time × velocity (*p* = 0.400), or condition × velocity (*p* = 0.527). In addition, there were no main effects for time (*p* = 0.158) or condition (*p* = 0.746), but there was a main effect for velocity (*p* < 0.001) (Figure 5). Time-to-peak torque decreased as angular velocity increased (*p* < 0.001).

### 3.5. Mean Power

**Figure 6:**
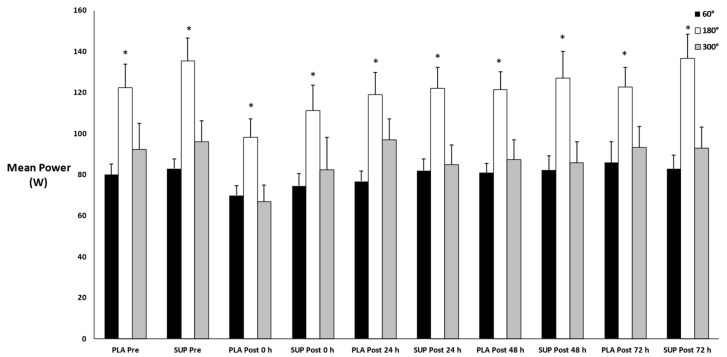
Mean ± SE of mean power. Pre: Pre-test. Post 0 h: Post-test 0 h. Post 24 h: Post-test 24 h. Post 48 h: Post-test 48 h. Post 72 h: Post-test 72 h. 60°: Velocity of 60${}^{\circ}\cdot s^{-1}$. 180°: Velocity of 180${}^{\circ}\cdot s^{-1}$. 300°: Velocity of 300${}^{\circ}\cdot s^{-1}$. * Denotes significant difference compared to 60${}^{\circ}\cdot s^{-1}$ and 300${}^{\circ}\cdot s^{-1}$.

There was no three-way interaction for time × condition × velocity (*p* = 0.487) and no two-way interactions for time × condition (*p* = 0.456), time × velocity (*p* = 0.319), or condition × velocity (*p* = 0.316). However, main effects for time (*p* = 0.033) and velocity (*p* < 0.001) were found. Post hoc analysis did not find any significant differences among the time points (*p* > 0.05) (Figure 6). In addition, there were significant differences between the velocities of 60°·s^−1^ and 180°·s^−1^ (*p* < 0.001), as well as between 180${}^{\circ}\cdot s^{-1}$ and 300${}^{\circ}\cdot s^{-1}$ (*p* < 0.001). However, there was no difference in mean power between 60${}^{\circ}\cdot s^{-1}$ and 300${}^{\circ}\cdot s^{-1}$ (*p* = 0.414). 

### 3.6. Muscle Soreness at Rest

**Figure 7:**
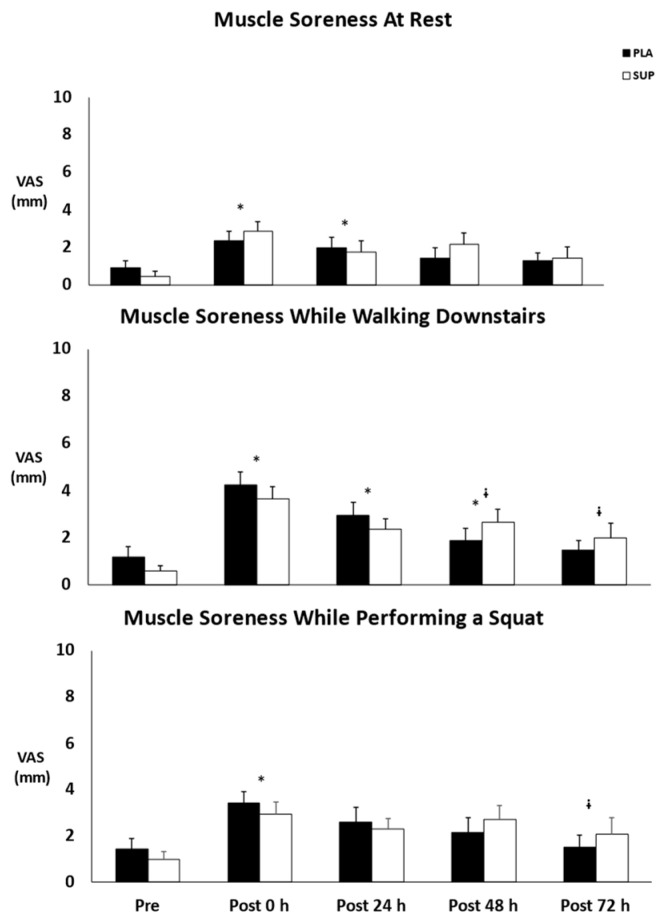
Mean ± SE of muscle soreness. Pre: Pre-test. Post 0 h: Post-test 0 h. Post 24 h: Post-test 24 h. Post 48 h: Post-test 48 h. Post 72 h: Post-test 72 h. PLA: Placebo. SUP: Supplement. * Denotes significant difference from pre-test. ᶤ Denotes significant difference from post-test 0 h.

There was no two-way interaction for time × condition (*p* = 0.082). In addition, there was no main effect for condition (*p* = 0.507), but there was a main effect for time (*p* < 0.001). Muscle soreness at rest increased from pre- to post-test 0 h (*p* < 0.001) and post-test 24 h (*p* = 0.039) (Figure 7).

### 3.7. Muscle Soreness While Walking Downstairs

There was no two-way interaction for time × condition (*p* = 0.063). In addition, there was no main effect for condition (*p* = 0.936), but there was a main effect for time (*p* < 0.001). Muscle soreness while walking downstairs increased from pre- to post-test 0 h (*p* < 0.001), post-test 24 h (*p* < 0.001), and post-test 48 h (*p* = 0.012) (Figure 7). There was also a decrease at post-test 48 h (*p* = 0.031) and post-test 72 h (*p* = 0.003) from post-test 0 h (Figure 7).

### 3.8. Muscle Soreness While Performing a Squat

There was no two-way interaction for time × condition (*p* = 0.147). In addition, there was no main effect for condition (*p* = 0.874), but there was a main effect for time (*p* < 0.001). Muscle soreness while performing a squat increased from pre- to post-test 0 h (*p* < 0.001) (Figure 7). There was also a decrease at post-test 72 h from post-test 0 h (*p* = 0.009) (Figure 7).

### 3.9. Rectus Femoris Muscle Activation 

**Figure 8:**
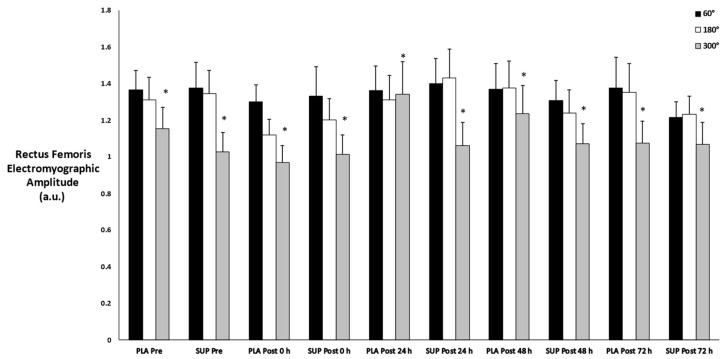
Mean ± SE of concentric rectus femoris muscle activation. Pre: Pre-test. Post 0 h: Post-test 0 h. Post 24 h: Post-test 24 h. Post 48 h: Post-test 48 h. Post 72 h: Post-test 72 h. 60°: Velocity of 60${}^{\circ}\cdot s^{-1}$. 180°: Velocity of 180${}^{\circ}\cdot s^{-1}$. 300°: Velocity of 300${}^{\circ}\cdot s^{-1}$. * Denotes significant difference compared to 60${}^{\circ}\cdot s^{-1}$ and 180${}^{\circ}\cdot s^{-1}$.

There was no three-way interaction for time × condition × velocity (*p* = 0.167) and no two-way interactions for time × condition (*p* = 0.866), time × velocity (*p* = 0.747), or condition × velocity (*p* = 0.466). However, there was a main effect for velocity (*p* = 0.005). For velocity, post hoc analysis found a difference in muscle activation between 60${}^{\circ}\cdot s^{-1}$ and 300${}^{\circ}\cdot s^{-1}$ (*p* = 0.029) and between 180${}^{\circ}\cdot s^{-1}$ and 300${}^{\circ}\cdot s^{-1}$ (*p* = 0.003) (Figure 8).

### 3.10. Vastus Lateralis Muscle Activation

**Figure 9:**
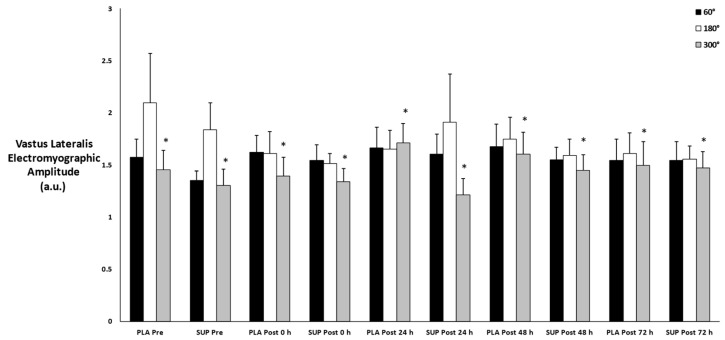
Mean ± SE of vastus lateralis muscle activation. Pre: Pre-test. Post 0 h: Post-test 0 h. Post 24 h: Post-test 24 h. Post 48 h: Post-test 48 h. Post 72 h: Post-test 72 h. 60°: Velocity of 60${}^{\circ}\cdot s^{-1}$. 180°: Velocity of 180${}^{\circ}\cdot s^{-1}$. 300°: Velocity of 300${}^{\circ}\cdot s^{-1}$. * Denotes significant difference compared to 180${}^{\circ}\cdot s^{-1}$.

There was no three-way interaction for time × condition × velocity (*p* = 0.495) and no two-way interactions for time × condition (*p* = 0.877), time × velocity (*p* = 0.200), or condition × velocity (*p* = 0.466). However, there was a main effect for velocity (*p* = 0.015). There was a difference in muscle activation between 180${}^{\circ}\cdot s^{-1}$ and 300${}^{\circ}\cdot s^{-1}$ (*p* = 0.024) (Figure 9).

## 4. Discussion

The primary purpose of this study was to investigate the effects of a tart cherry supplement on recovery from an exhaustive bout of exercise. The primary results indicated there were no supplement-related differences in isokinetic muscle peak torque, peak power, total work, time-to-peak torque, mean power, muscle soreness, or muscle activation between the supplement and placebo conditions. One of the major differences between the current study and previous research is the quantity and form of the tart cherry supplement used. In this present study, the tart cherry supplement was in capsule form and contained 1000 mg total of concentrated tart cherry extract per serving. One of the first studies to report the efficacy of tart cherry in attenuating strength loss following muscle damage had participants supplement with a 12 oz bottle of cherry juice blend with at least 600 mg of phenolic compounds and at least 40 mg of anthocyanins [26]. Another study demonstrated that 10.5 oz of tart cherry juice containing at least 600 mg of phenolic compounds and 40 mg of anthocyanins reduced muscle pain during long distance running [31]. Brown et al. [28] reported that a cherry juice blend containing a 30 mL dose of concentrate with a total anthocyanin content of 73.5 mg L^−1^ of cyanidin-3-glucoside, a total phenolic content of 178.8 gallic acid equivalent L^−1^, and an antioxidant capacity of 0.58 trolox equivalents L^−1^ attenuated the symptoms of muscle damage and improved recovery among women. Another study indicated that supplementation with a 500 mg proprietary broad-spectrum tart cherry powder with total polyphenols of 5–6% *w*/*w* tested via an F-C assay reduced the markers of oxidative stress, skeletal and cardiac muscle damage [2]. One study, however, reported that supplementation with 60 g of tart cherry powder mixed with unsweetened Black Cherry Kool-Aide—which provided 64 mg of anthocyanins and 733 mg of phenolic compounds—resulted in no improvements in recovery [3]. Overall, the differences in polyphenol and anthocyanins, as well as the form in which the tart cherry was consumed, could explain some of the differences in the results. Although the dosage in the present study was higher than in earlier studies investigating tart cherry supplements it is possible that the lack of EIMD in the current overload protocol did not allow for any improvements to be observed.

In the present study, there was no condition-specific difference in peak torque or time-to-peak torque. The present results were inconsistent with those of Connolly et al. [26], which demonstrated that supplementation with a placebo for eight days resulted in greater loss in isometric elbow flexion strength compared to cherry juice following an eccentric exercise protocol on the fourth day of supplementation in college-aged males. Beals et al. [3], however, reported no significant difference in isokinetic concentric quadriceps strength between a tart cherry blend group and a placebo group of recreationally active men and women who consumed the supplement for four days before and eight days following an exhaustive bout of eccentric exercise. Similar to the previous studies [3,26], the participants in the present study consumed either the placebo or the tart cherry supplement acutely before, during, and after the exercise protocol when conducting the post-testing. Previous research has shown that supplementation with polyphenols daily for 3 or more days prior to and following exercise may improve recovery [26]; however, the results of the current study failed to show improvements in muscle function after supplementation for 4 days before and after an overload protocol. Decreases in CK suggest that antioxidant supplements may be able to reduce the amount of biomechanical damage placed on muscle proteins by scavenging for ROS [2]. Since the present study did not measure the blood markers of muscle damage, it is unclear whether there were any improvements in damage to the muscle proteins. At least in terms of muscle function—particularly peak torque and time-to-peak torque—no improvements were observed.

The findings of the present study indicated no condition-specific difference in peak power and mean power measured via an isokinetic dynamometer. Hillman et al. [6] used a countermovement jump (CMJ) to assess muscle power recovery following 5 sets of 10 drop jumps among men and women who supplemented with either a tart cherry and whey beverage or a placebo and found no differences in CMJ over time or between groups. It was suggested that the use of the CMJ may not have been sensitive enough to evaluate neuromuscular performance [6]. Moreover, McCormick et al. [37] used a vertical jump (VJ) as a performance variable in a study examining the effects of tart cherry juice compared with a placebo on recovery following a simulated fatiguing team game activity in well-trained male water polo players and found no differences between conditions, as well as pre-testing to post-testing performance. Hooper et al. [2] also indicated no significant difference in VJ performance following 6 sets of 10 repetitions of a barbell back squat at 80% of 1RM between a placebo versus a tart cherry supplement condition, although it was trending toward significance. In contrast, Brown et al. [28] reported that supplementation with tart cherry improved recovery in CMJ after a repeated-sprint protocol of 15 × 30 m maximal sprints with a rapid 10 m deceleration phase compared to a placebo among physically active females. It is possible that the different muscles being trained and their typical load volume influenced the potential soreness and performance decreases reported in the previous studies [2,6,28,37]. Furthermore, it is likely that the overload protocol used in the present study did not induce sufficient fatigue in the lower limbs to cause performance decreases. 

The present study found no difference between conditions for total work, but total work did decrease from pre-test to post-test 0 h before increasing at the subsequent post-tests of 24 h, 48 h, and 72 h at the three velocities. These results are consistent with those of Botwell et al. [4], who reported no differences in relative work between a tart cherry trial and a placebo trial. Specifically, Botwell et al. [4] analyzed biomechanical recordings in well-trained male participants who completed 10 sets of 10 single-leg knee extensions at 80% of their 1RM with a 3 s elongated eccentric phase after supplementation with either cherry juice or a placebo for 10 days. Work—one of the biomechanical recordings—was determined by integrating the force–time trace, and data were normalized to the corresponding 1RM value to eliminate inter-individual and inter-leg variability [4]. Due to the uniqueness of total work as a performance measure representing an entire set rather than individual repetitions, more research is needed using total work as a marker of recovery.

The present study indicated that muscle soreness assessed at rest, while walking downstairs, and while performing a squat increased from pre-test to post-test 0 h; however, the reported values were not high and perhaps influenced the lack of significant results for the supplement condition. Hillman et al. [6] and Quinlan and Hill [8] also measured muscle soreness in the lower body by having participants perform a squat and reported increased muscle soreness over time with no significant group effects following 5 × 20 drop jumps and an adapted version of the Loughborough Intermittent Shuttle Test (LIST), respectively. Furthermore, Hooper et al. [2] had participants report their muscle soreness using a marked line on a 10 cm scale as a researcher firmly palpated their upper, middle, and lower quadriceps while in a seated position with their legs elevated following 6 sets of 10 repetitions of barbell back squats at 80% of 1RM. The results of Hooper et al. [2] demonstrated increased muscle soreness over time but no condition-specific difference. To reduce the subjectivity associated with using a VAS, Botwell et al. [4] opted to use pressure pain threshold on the belly of the rectus femoris, vastus lateralis, and vastus medialis to measure muscle soreness but found no significant difference between a cherry juice concentrate and an isoenergetic fruit concentrate placebo. In contrast, Connolly et al. [26] reported that a placebo trial had significantly higher pain values (reported as the overall discomfort during active elbow flexion and extension in activities of daily living) following 40 maximal eccentric contractions of the elbow flexors compared to a cherry juice trial. In addition, the pain values peaked at post-test 48 h for the placebo trial, whereas the pain values peaked at post-test 24 h for the cherry trial [26]. It is possible that the differences in results may be due to the different muscles being assessed, exercise protocols, and the manner in which muscle soreness was measured.

Previous research on semi-professional male soccer players reported no decline in MVC (measured using a strain gauge) of the dominant knee extensors in a tart cherry supplementation group compared to a placebo group, whose MVC did not return to basal levels at post-test 72 h [25]. In addition, Botwell et al. [4] reported that knee extension isometric MVC force (normalized to pre-exercise values) recovered significantly faster with tart cherry supplementation. In contrast, Brown et al. [28] reported no difference in the recovery of isometric MVC of the right knee extensors among physically active females consuming a tart cherry supplement or a placebo for four days prior to a muscle damaging protocol, on the day of the protocol, and three days after the protocol. While these studies [4,25,28] used isometric MVC to assess muscle function, the present investigation was unique because it was one of the first to use EMG to analyze muscle function following tart cherry supplementation. Specifically, the current study found no difference in RF and VL EMG muscle activation between conditions. It is possible that previous training of the participants may have influenced the results. For example, although the participants were recreationally active, some of them may have had previous resistance training experience, which may have attenuated their response to a bout of exhaustive exercise compared to those who have had no experience with resistance training. 

One limitation of this study was the lack of oxidative stress biomarkers. Therefore, it is unclear whether those measures would have revealed different results. However, a variety of isokinetic measures more reflective of performance were analyzed. Additionally, the population used may have had limited exposure to maximal effort exercise training, such that the results may be different when investigating the effects on resistance trained women or athletes compared to recreationally active females. Another limitation of this study is the lack of menstrual cycle tracking and consideration during testing. It has been suggested that EIMD and recovery may vary throughout the different phases of the menstrual cycle [28]. Lastly, based on the low muscle soreness values reported and the peak of muscle soreness occurring at post-test 0 h, it is possible that the overload protocol used in this study did not induce enough exercise-induced muscle damage for the supplement to have an effect. Future research could follow a similar protocol to investigate the differences in women who meet specific strength requirements. Another future alternative could involve a comparison of the effects of tart cherry supplementation on performance and recovery between men and women. Future research could also investigate the effects of tart cherry supplementation using a loading phase of fewer than three days.

## 5. Conclusions

The primary results of the present study indicated no supplement-related differences in isokinetic muscle peak torque, peak power, total work, time-to-peak torque, mean power, muscle soreness, or muscle activation between the supplement and placebo conditions. In summary, compared to a placebo, tart cherry taken four days before, on the day of, and three days after an exhaustive bout of exercise did not demonstrate reduced attenuation of muscle function or muscle soreness and muscle activation of the quadriceps.

## Data Availability

Data will be made available upon reasonable request.
